# Editorial for the Special Issue on “Geometry Reconstruction from Images”

**DOI:** 10.3390/jimaging10020029

**Published:** 2024-01-23

**Authors:** Daniel Meneveaux, Gianmarco Cherchi

**Affiliations:** 1 XLIM Institute, UMR CNRS 7252, University of Poitiers, 86073 Poitiers, France; 2 Department of Mathematics and Computer Science, University of Cagliari, 09124 Cagliari, Italy

This special issue on geometry reconstruction from images has received much attention from the community, with 10 published papers. Despite the numerous existing approaches in the field, this scientific area still motivates numerous researchers, and many challenges remain.

The contribution proposed in [1] concerns a method dedicated to the identification and 3D reconstruction of fishes from structured light. The projected patterns are employed to determine fish species, thus allowing identification, classification, and 3D reconstruction. This novel approach, though specifically devised for fishes, may also provide further methodologies for other types of applications.

Reconstruction of floorplans from point cloud images has been proposed in [2], using generative adversarial networks. The approach consists of generating masks that represent room layouts associated with a repairing process and edge optimization or geometric refinement.

Another interesting approach has been devised for managing contour appearance [3]. The goal is to modulate the contours of existing shapes, given geometric frequency, amplitude, and waveform. The authors aim to preserve a homogeneous visual appearance of the deformation.

The question of image acquisition in various environments and the associated distortions has been addressed in [4]. The authors propose to study the radial distortions and formulate a correction term for improving image characteristics and thus recovering a correct geometry.

Managing geometry with images also requires managing clipping, as explained in [5]. The authors propose an overview of line clipping algorithms against rectangular windows, with comparisons and recent contributions.

An illustration of practical 3D reconstruction is made in [6], with bottle caps detection. The authors study cylindrical distortions that may impact the reconstruction process and propose an acquisition process based on a four-camera system.

From a set of points lying on a sphere, detecting and identifying a series of circles has been studied in [7]. The authors identify and classify subsets of circular points. Such a study is of great interest for road-line detection, geophysics, or spherical tesselation.

In [8], the authors propose improving the geometric precision of 3D reconstruction from medical images thanks to a combination of the Marching Cubes algorithm and smooth implicit curve tracking. The proposed approach produces a space partition (without holes) that ensures 3D consistency.

The problem of 3D reconstruction from a single image is addressed in [9] with a survey of methods that specifically rely on deep learning approaches. The review illustrates the results of reconstructing 3D shapes as depth maps, surface normals, point clouds, and meshes. It also introduces loss functions and metrics dedicated to the training and evaluating the studied methods.

Studying the registration of 3D scanned objects from images is also challenging when the observed morphology changes according to the viewpoint [10]. In this paper, the authors study the registration error according to the field of view and propose guidelines for improving the acquisition process.

All these exciting contributions demonstrate the scientific dynamism of the area. They have motivated the opening of a second special issue on this subject in order to encourage other authors to submit their original research.

## References

[1] Veinidis C., Arnaoutoglou F., Syvridis D. (2023). 3D Reconstruction of Fishes Using Coded Structured Light. J. Imaging.

[2] Jin T., Zhuang J., Xiao J., Xu N., Qin S. (2023). Reconstructing Floorplans from Point Clouds Using GAN. J. Imaging.

[3] Presnov D., Kolb A. (2022). Perception and Quantization Model for Periodic Contour Modifications. J. Imaging.

[4] Senshina D., Polevoy D., Ershov E., Kunina I. (2022). Experimental Study of Radial Distortion Compensation for Camera Submerged Underwater Using Open SaltWaterDistortion Data Set. J. Imaging.

[5] Matthes D., Drakopoulos V. (2022). Line Clipping in 2D: Overview, Techniques and Algorithms. J. Imaging.

[6] Zhu X., Liu Z., Zhang X., Sui T., Li M. (2022). A Very Fast Image Stitching Algorithm for PET Bottle Caps. J. Imaging.

[7] Ibrahim B., Kiryati N. (2022). Detecting Cocircular Subsets of a Spherical Set of Points. J. Imaging.

[8] Lechelek L., Horna S., Zrour R., Naudin M., Guillevin C. (2022). A Hybrid Method for 3D Reconstruction of MR Images. J. Imaging.

[9] Khan M.S.U., Pagani A., Liwicki M., Stricker D., Afzal M.Z. (2022). Three-Dimensional Reconstruction from a Single RGB Image Using Deep Learning: A Review. J. Imaging.

[10] Benfield K.J., Burruel D.E., Lujan T.J. (2023). Guidelines for Accurate Multi-Temporal Model Registration of 3D Scanned Objects. J. Imaging.

